# Multifaceted Effects of Lycopene: A Boulevard to the Multitarget-Based Treatment for Cancer

**DOI:** 10.3390/molecules26175333

**Published:** 2021-09-02

**Authors:** Stefania Marzocco, Rajeev K. Singla, Anna Capasso

**Affiliations:** 1 Department of Pharmacy, University of Salerno, 84084 Fisciano, Italy; annacap@unisa.it; 2 Institutes for Systems Genetics, Frontiers Science Center for Disease-Related Molecular Network, West China Hospital, Sichuan University, Chengdu 610041, China; rajeevsingla26@gmail.com; 3 iGlobal Research and Publishing Foundation, New Delhi 110059, India

**Keywords:** antioxidants, antitumor, carotenoids, lycopene

## Abstract

Lycopene is a pigment belonging to the group of carotenoids and it is among the most carefully studied antioxidants found especially in fruit and vegetables. As a carotenoid, lycopene exerts beneficial effects on human health by protecting lipids, proteins, and DNA from damage by oxidation. Lycopene is a powerful oxygen inactivator in the singlet state. This is suggestive of the fact that lycopene harbors comparatively stronger antioxidant properties over other carotenoids normally present in plasma. Lycopene is also reported to hinder cancer cell proliferation. The uncontrolled, rapid division of cells is a characteristic of the metabolism of cancer cells. Evidently, lycopene causes a delay in the progression of the cell cycle, which explains its antitumor activity. Furthermore, lycopene can block cell transformation by reducing the loss of contact inhibition of cancer cells. This paper collects recent studies of scientific evidence that show the multiple beneficial properties of lycopene, which acts with different molecular and cellular mechanisms.

## 1. Introduction

Lycopene is an acyclic linear carotenoid characterized by eleven conjugated double bonds. Unlike β-carotene, it is not transformed into vitamin A in the body. Lycopene exists in several stereoisomeric forms. Double bonds are subject to isomerization. In nature, lycopene is found in the structural form of “trans”-type isomers; however, exposure to heat sources or even light irradiation involves a modification of its structure in cis isomers (mainly in positions 5, 9, 13, and 15), which are more assimilable by the human body, showing, therefore, a greater bioavailability. Quite possibly this might also occur in vivo. Various lycopene forms are slightly soluble in water. For example, lycopene is found either in crystalline form in the chromoplast or it is available in the form of a carotenoid-protein complex in chloroplasts. Thermal processing enhances the bioavailability of lycopene as it disrupts cellular membranes, which leads to the escape of lycopene from the tissue matrix. Therefore, processed food that involves the concentration procedure, which is associated with water loss, certainly contributes to making cooked tomatoes a great reservoir of lycopene compared to the raw product. Likewise, cooked carrots serve as a great source of carotene [1].

Lycopene is a thermo-stable carotenoid and cooking does not damage it; on the contrary, heat can make the molecule even more bioavailable, i.e., more assimilable by the body. Thus, cooking food can increase lycopene bioavailability and this could be credited to the dissociation of lycopene containing complex proteins [1]. Another reason could be the dispersion of carotenoid aggregates due to the cooking, which are usually crystalline.

Numerous studies suggest that a high consumption of tomatoes decreases the contraction rates of cancer. Given that the tomato is one of the most consumed vegetable products in the world, it is extremely interesting to note these results. Based on several investigations, lycopene seems to exert a preventive action, among other things, against prostate cancer [1,2].

Many epidemiological studies confirm this last thesis, correlating the increase in the consumption of lycopene through the diet and the decrease in the risk of prostate cancer. These results are strengthened by substantial scientific evidence that in addition to antioxidant activities, lycopene can cause inhibition of proliferation and apoptotic induction, and impair the metastatic potential of cancerous cells in the prostate gland [3]. In this review we analyze the possible effects of lycopene on the different aspects and steps of tumor development and progression.

## 2. The Antioxidant Properties of Lycopene

### 2.1. Molecular Mechanisms

Oxidative stress is due to an increased production of reactive oxygen and nitrogen species (ROS, RNS), which are not sufficiently balanced by antioxidant cellular systems. These species include the hydroxyl radical (OH), the radical superoxide (O_2_), peroxynitrite (ONOO), peroxyl (ROO), hydrogen peroxide (H_2_O_2_), singlet oxygen (^1^O_2_), and ozone (O_3_) [4].

A myriad of cellular processes, such as inflammation, ischemia or reperfusion, metabolic activities, and mitochondrial respiration, generate these chemical species [5]. The uncontrolled production of these chemical species may cause significant cellular damage due to the oxidation of cellular biomolecules such as DNA, proteins, and lipids. Consequently, this might augment processes related, for example, to carcinogenesis, cell transformations, resistance to apoptosis, proliferation, metastasis, angiogenesis, DNA damage, and therefore mutations, as well as genetic instability [6].

Lycopene possesses consistent antioxidant activity, which is exerted through various mechanisms [3]. Lycopene is an alluring biological target for electrophilic reagents, credited to its electron-rich structure. Further, the electron-rich reservoir bestows a remarkable oxygen as well as free radical reactivity upon lycopene. Quite aptly, lycopene tops the list of carotenoids in terms of oxygen quenching ability and also offers the possibility to interfere in free-radical-initiated reactions, such as ROS [7]. It is speculated that the antioxidant properties of lycopene are responsible for its cancer inhibitory roles and prevention of various chronic ailments [8].

Lycopene and other carotenoids act as antioxidants via different mechanisms. The reactive oxygen species (for example, “singlet oxygen” (^1^O_2_) (highly reactive), which can oxidize nucleic acids, unsaturated fatty acids, or amino acids) can be neutralized by carotenoids/lycopene by carrying out the following reaction:

^1^O_2_ + LYC→^3^O_2_ + ^3^LYC

^3^LYC→LYC + heat

The higher amount of energy that lycopene has obtained in this reaction, reaching the triplet state, is then delivered through vibrational as well as rotational interactions with solvents, and with the consequent liberation of thermal energy. Again, the extended conjugated polyene lycopene structure is accountable for the above reaction. Once the molecule reaches its ground state, another ^1^O_2_ can be neutralized, thus providing the ability for each carotenoid molecule to extinguish approximately one thousand molecules of ^1^O_2_ [8].

#### 2.1.1. Modulatory Effect on Lipid Peroxidation and DNA Damage

Lycopene along with the carotenoids are well-known for their antioxidant activity aimed at preventing free radical reactions. During the lipid peroxidation process, the peroxyl radicals are fortified in the body. Eventually, this might cause damage to the lipophilic sections. Further, the amelioration of such highly reactive species accentuates the formation of radical adducts. Collectively, these crucial oxidation products of lycopene partake in the membrane repair process via lipid peroxidation [8,9].

Given the above result, the study by Matos et al. [10] reports the ability of lycopene to protect mammalian cells from an iron chelator-induced lipid peroxidation and oxidative DNA damage in vitro chelator. Besides this, the damage to mitochondrial DNA caused by the production of ROS through UV radiation is partially abrogated by lycopene tomato sauce in vivo [11,12].

#### 2.1.2. Modulation of Antioxidant Responsive Elements (ARE) and Nrf2

Several studies have shown the effects of lycopene on the induction of antioxidant enzymes and detoxifying enzymes of phase II. In vivo studies performed by Bhuvanewari et al. suggested that lycopene (2.5 mg/kg) can potentially suppress gastric cancer by multimodal mechanisms of reduction in lipid peroxidation, elevation in the levels of antioxidants, and enhanced GSH-dependent enzyme activities; for example, glutathione reductase, glutathione peroxidase, and glutathione-s- transferase [13,14].

Nrf2 (nuclear factor E2-related factor 2) is an important ARE (antioxidant response element), which is integral to the reactions involved in detoxification of carcinogens and antioxidant cell defense system modulation. This is because it promotes the upregulation of phase II cytoprotective enzymes induced by stress. Depending on this, Nrf2 also produces anti-inflammatory effects [15].

Several pieces of evidence suggest that lycopene is capable of upregulating electrophilic antioxidants and antioxidant responsive elements (EpRE/ARE) and, again, nuclear factors (Nrf2), generating the production of detoxifying-antioxidant enzymes (phase II). These, in turn, provide protection to the cells against various reactive oxygen species as well as electrophilic molecules [16,17,18]. The upregulated transcription of the genes coding for antioxidant enzymes and detoxifying phase II occurs via DNA sequences present in promoter regions of AREs. Reportedly, lycopene is known to “upregulate” this ARE system via Nrf2 in vitro (HepG2 and MCF-7 cells) [15].

In general, Nrf2 is localized in the cytoplasm, where it specifically binds to the inhibitory protein known as Keap1 and forms a complex. Although this Nrf2 and Keap1 complex is dissociated under the conditions of oxidative stress, the rescue of Nrf2 from proteasome degradation, together with the induction of translocation of Nrf2 itself to the nucleus, enables its binding to the AREs along with various other transcription factors. This allows the regulation of gene expression of detoxifying/antioxidant enzymes, such as Heme oxygenase and NAD(P)H quinone oxidoreductase 1 [19,20,21].

The molecular mechanisms underlying lycopene-mediated induction of Nrf2 nuclear translocation are scarcely known. Lian and Wang [16] hypothesized that the highly reactive aldehyde groups present in lycopene metabolites facilitate Schiff base formation with the group N-terminus of proteins. Specifically, it can cause a direct modification of the cysteine residues in Keap1, then repeal ubiquitination followed by Nrf2 degradation mediated by Keap1. Next, there occurs an oxidation of covalent modification of thiol groups, which are present in the Keap1 harbored cysteine residues. Finally, these steps allow Nrf2 and Keap1 complex dissociation [22,23].

Quite possibly, these “lycopenoids” influence upstream signaling pathways. Precisely, these lycopenoids target the receptors for epidermal growth factor (known as EGFR), mitogen-activated protein kinases (MAPKs), phosphoinositide 3-kinase(PI3K) and protein kinase C (PKC). In addition, Nrf2-ARE regulating proteins and those involved in pulmonary epithelial cell signal regulation are targeted by these lycopenoids [24].

#### 2.1.3. Expression Modulation of P450

The cytochrome P450 family is an enzymatic superfamily of proteins present in all domains of living beings and is involved in the detoxification of the organisms, being able to act on a large number of different substrates. It also partakes in the metabolism (usually oxidative) of myriad lipophilic compounds that are of endogenous or exogenous origin. Since the P450 cytochrome catalytic cycle is poorly coupled, there occurs steady and uninterrupted ROS production. Consequently, the pathways for cellular signaling and associated functions are affected [25,26,27].

It is well-documented that carotenoids cause an induction of cytochrome-associated (P450 family) enzymatic activities [28,29]. Notably, Astorg et al. [30] put forward the hypothesis that lycopene exerts its protective effect against preneoplastic lesions by modulating cytochrome P450 2E1 enzymes, when studied in a rat model of a tumor. Furthermore, lycopene administration in rat tumor models induced hepatic cytochromes in a dose-dependent manner. These specifically included cytochromes 1A1/2, 2B1/2 and 3A [31]. The observations that the activity of the P450 was induced by plasma levels of lycopene indicate that carotenoid-mediated modulation of the metabolism of these could plausibly be of considerable relevance to humans. There are no human data suggesting that P450 upregulation is effective. 

#### 2.1.4. Inhibition on iNOS and COX-2 Expression

Lycopene counters iNOS (inducible nitric oxide synthase) effects, for example through inhibiting the production of nitric oxide (NO). The effects of lycopene as well as quercetin and tyrosol (natural antioxidants), gene expression of iNOS, and cyclooxygenase-2 (COX-2), have been studied in vitro. In particular, the gliadin and interferon-gamma (INF-γ)-stimulated macrophage cell line (RAW 264.7) was used, wherein this combined therapeutic strategy was able to reduce iNOS and COX-2 expression. Lycopene can therefore decrease the gene expression of iNOS and COX-2 as a non-toxic agent via controlling pro-inflammatory genes [32]. This mechanism is also supported by another study, which was performed by Rafi et al. They found that 10 μmol/L of lycopene was capable of reducing the lipopolysaccharide (LPS)-induced NO production by approximately 40% when compared to a control in the RAW 264.7 mouse macrophage cell line. They also claimed that the lycopene caused a decline in the LPS-induced protein and mRNA expression of iNOS, after performing Western blotting and RT-PCR expression analysis as previously described [33].

#### 2.1.5. Downregulation of NF-kB Modulation

Evidently, NF-kB is the first among the transcription factors that is also activated by oxidative stress in eukaryotes. This is accredited to the following mechanisms: the first involves the improvement of the ROS-mediated degradation of IkB, while the second leads to the oxidative improvement of the upstream signal cascade [34,35,36].

It is well-documented that lycopene inhibits NF-kB binding activity and target gene expression, especially of NF-kB and MMP-9-associated gene targets, which prohibits the cell invasion of hepatoma in humans. This inhibition occurs when IkB phosphorylation and expression of NF-kB are downregulated, also due to p56 subunit nuclear translocation [37].

The LPS (lipopolysaccharide) stimulation activates the MAPK signal path along with NF-kB. Lycopene treatment significantly inhibits NF-kB, p-ERK, p-JNK, and p-p38 upregulation induced by LPS [38].

## 3. The Antineoplastic Properties of Lycopene

**Figure 1:**
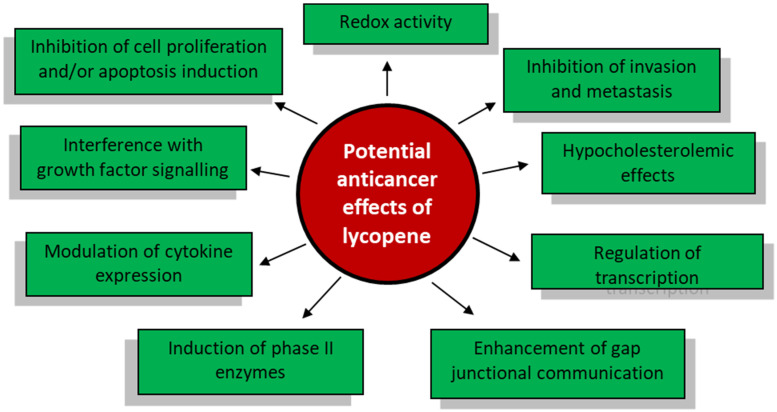
Schematic representation of potential anticancer effects of lycopene.

The potential cancer prevention mechanisms of lycopene are summarized in Figure 1 [39,40].

Oxidative stress is among the key contributing factors associated with an enhanced cancer-risk [41]. Many works, some of which were cited in the previous chapter, have been directed toward characterizing the functional attributes of lycopene with a focus on its antioxidant properties. Reportedly, lycopene molecules neutralize the energy of various inimical oxygen forms (e.g., singlet oxygen) and, in turn, “cleanse” a gamut of free radicals efficiently owing to the conjugated double-bond systems contained within [42].

The ability of lycopene to inhibit cancer cell proliferation is observed in different cancer cell types. The inhibitory effect is followed by the abrogation of progression of the cell cycle from G0/G1 to S phase and the variation of proteins that control the cycle [43]. Lycopene, in particular, causes a decline in levels of cyclin D1 with a corresponding elevation in p53, p21 proteins in cancerous cells. The induction of apoptosis is due to the modulation of the expression and/or phosphorylation, by lycopene as well as other carotenoids, of proteins like Bcl-2, Bad, Bid, and Bax [44].

There is accumulating evidence that lycopene may modulate IGF-1, which causes a reduction in growth in cancer cell lines [44]. Sharoni et al. [45] predicted a possible mechanism that enables lycopene to interfere with the cell growth stimulated by IGF-1. This study demonstrated that a physiological concentration (estimated to be about 0.7 µmol/L) of lycopene reduced the cell growth stimulated by IGF-1 in endometrial, breast, and lung cancer cells.

The phase II enzymes upon induction confer protection against myriad animal and human carcinogens. These are important antioxidants involved in conjugating reactive electrophilic species [39].

Transcription, the process by which information contained in DNA is enzymatically transcribed into a complementary RNA molecule, can be regulated by lycopene; expression alterations of different proteins is suggestive of the fact that lycopene primarily modulates transcription [44,45].

Cancer cells display abnormally altered cholesterol biosynthetic pathways. These are also resilient to downregulation of cholesterol, and farnesylation (post-transcriptional modification of proteins by which an isoprenyl group is added to a cysteine residue). Notably, this is indispensable in the activation of oncogenes [46]. In different tumor cell lines, a dose-dependent lycopene treatment reduces intracellular levels of total cholesterol and decreases HMG-CoA (hydroxymethylglutaryl-coenzyme A) [39,47]. Chen and Huang also discussed the importance of fatty acid metabolism in cancer. They mentioned that modifications in lipid metabolism, especially the synthesis of fatty acids as well as their uptake in the cells, play a critical role in metabolic reprogramming in cancer cells, which then exerts a supportive action for cell proliferation, growth, and dissemination [48].

Reportedly, pro-inflammatory cytokines including interleukins or TNF-α (tumor necrosis factor) promote tumors as observed in different experimental models of carcinogenesis [49,50]. The potency of lycopene to impact the level of cytokines is likely to be partly due to the location of carotenoids (in or within the cell membranes), modulating the molecules at the surface for primary immune response, factor transcription, and production of ROS, etc. [51].

The literature has reported that carotenoids and retinoids stimulate the GJC (gap junction communication) by stabilizing connexin 43 mRNA [44]. This property is considered chemopreventive in cancer cells. The lycopene as well as its products of cleavage were evaluated for their effects on GJC, connexin 43 mRNA stabilization, and RAR-β2 promoter transactivation in vitro [52], thus also supporting lycopene effects on GJC.

A critical feature of cancer cells (metastatic type) is their ability to dissolve basal membranes and extracellular matrix with the aid of matrix metalloproteinases (MMPs) [53,54]. In one study, Hwang and Lee [55] showed that lycopene may decrease the activity of metalloproteinases of the matrix and prevent SK-Hep1 cellular adhesion, invasion, and migration. In addition, lycopene-mediated induction of the metastasis suppressor gene nm23-H1 was observed [55,56].

### 3.1. Effect on Cancer Cell Proliferation and Growth

Carotenoids are being studied concerning one of their effects, namely antineoplasticity. In particular, a study conducted by Kotake-Nara et al. [57] evaluated various carotenoids (15 in total), focusing on the growth-inhibiting effect in vitro. This included prostate cancer cell lines; namely, DU145, PC3, and LNCaP. A significant reduction in cellular viability after a treatment with 20 µmol/L of acyclic carotenoids was observed. Treatment at concentrations of 5 µmol/L was effective in increasing cell viability reduction, especially in the case of lycopene. Another study, in which tomato concentrate extract was used, found a decrease in the proliferation of LNCaP cells (in a time and dose-dependent fashion), with a maximum effect noted in the presence of 5 µmol/L lycopene. On expiry of the incubation period (48 h), cellular growth was inhibited by 67%. However, given the use of tomato concentrate extract instead of pure lycopene, probably the synergistic effects may come into play [58].

### 3.2. Molecular Mechanism of Lycopene on Prostate Cancer Cells

Cancer of the prostate gland ranks second in cancer-associated mortality in the US [59,60]. In fact, it is related with the most frequent cancer-related mortality in the male population, also at a global level [61]. There is substantial epidemiological evidence on the inverse relation between tomato consumption or the edible products derived from tomatoes and susceptibility to prostate cancer [62]. Many studies on prostate cancer cells have reported that lycopene can serve as an anticancer agent by stopping proliferation of cells and/or apoptotic induction. This beneficial effect in other organs is still under discussion [3]. Tang et al. [63] found insignificant inhibition on the lycopene-treated DU145 cells for the initial 24 h. In this study, the concentrations of lycopene were above 50 µmol/L. A significant inhibition, however, was always observed in lycopene-treated DU145 cells, but from 48 to 96 h. Lycopene at a concentration higher than 20 µmol/L after 96 h significantly inhibits DU145 cells as compared to the control. IC_50_ for lycopene against the DU145 cell line was 26.6 µmol/L at 96 h. However, this concentration is not physiologically observed in normal instances, and the current findings show a reduction of 10 percent in cell viability post 96 h with concentrations of 1, 3, and 5 µmol/L.

A plausible explanation linked to the development of cancer of the prostate gland comprises several genetic mutations that control cellular differentiation and growth [64]. A chronic inflammatory process, for example in bacterial infection of the prostate gland or prostatitis, might perhaps be linked to ROS production. This can cause oxidative DNA damage via genetic mutations. Exogenous inducers of carcinogenesis might present a similar effect. DNA aberrations are, in general, controlled by the cellular surveillance system that intervenes by blocking the cell cycle initiator cells to restore integrity of DNA. In case the damage to the DNA is beyond repair, cellular apoptosis would be one of the possible outcomes. However, a malfunction of the repair mechanism may induce mutations, followed by cancer [65].

A study showed that ROS elevation in cancer cells in the prostate has been linked with androgens. Goo et al. then investigated, by carrying out a quantitative proteomic analysis, the alterations in the expression of proteins in LNCaP cells of the decrease-androgen and the sufficiency-androgen [66].

In addition, it has been observed that lycopene is capable of inducing a modest elevation of all these proteins detoxifying in LNCaP cells that are “androgen-rich”, and a significant increase to those in “androgen-deficit”. For this reason, it is conjectured that lycopene executes a DNA damage prevention mechanism precisely because of its ability to increase the number of detoxifying proteins [67].

Another study analyzed the expression of proteins in lycopene-treated (2 µmol/L, 48 h) prostate epithelial cells using proteomics methods (iTRAQ) [68]. Lycopene enhanced the production of phase II protective enzymes viz. glutathione-S-transferase-omega-1, oxidoreductase sulfide-quinone, and peroxiredoxin-1. Furthermore, proteins including ERO1 and CLIC-1, generally involved in the formation of ROS, have been decreased after lycopene treatment. This indicates the ability of lycopene to minimize ROS formation and mitigate oxidative stress [3].

Lycopene activity in clinical use is supported by randomized clinical trials indicating lycopene’s ability to decrease PSA-levels and to affect proliferating prostate cells in patients affected by prostate hyperplasia or cancer [3]. Despite this, new randomized clinical trials would contribute to better understanding and elucidating the effect of lycopene in prostate-cancer-affected patients.

### 3.3. Effects of Lycopene on the Cell Cycle

Epidemiological and clinical studies suggest evidence of a plausible protective effect of lycopene: the mechanism of this action includes the cell cycle arrest and apoptotic induction.

Lycopene negatively influences the cancer cells directly, or cancer development via modulation of cell cycle commencement and proliferation of cells. This process displays the inhibitory effect on the synthesis of DNA, on the opening of the upregulation protein gap-junction, and local androgenic signal reduction, and has an impact on the signal IG F-1 activity antioxidant and on the cell death induction by apoptosis, suggesting the chemopreventive potential of carotenoids with diverse genomic and non-genomic cellular effects [3,69,70].

Most cell-cycle studies have been performed on lycopene-treated (post 48 h) breast and prostate cancer cell lines. There are considerable data on lycopene-induced cell-cycle arrest [43]. Recent studies have shown that the growth of cells in human hepatoma is inhibited by 20–50 percent by lycopene in a low physiological concentration of 0.2 pmol/L. Evidently, lycopene induces the arrest in the G0/G1 phase and is blocked at stage S in cell lines with prostate cancer (LNCaP and PC3) [71].

Moreover, the lycopene-induced inhibition of cell growth (MCF-7 cells) corresponded to a decreased expression of c-Myc and Cyclin D. In particular, with the reduced activity of CDK4 and p27 maintenance within the complex, cyclin E-CDK2 leads to a diminished kinase CDK2 activity, with the consequent reduction in phosphorylation of Rb and therefore inhibition of a transition G1/S [72].

There are further papers demonstrating the ability of lycopene to block cells in the G1/S transition phase thanks to lowered cyclin D and elevated p21, p27, and p53 levels in LNCaP [39]. Lycopene prompts these cellular alterations via Ras inactivation. Ras is inactivated through mevalonate abrogation and reduces HMG-CoA expression. Treatment with lycopene also retards Ras farnesylation, which facilitates Ras accumulation in the cytoplasm and its inactivation ensues. Besides, lycopene downregulates the Ras-dependent activation of the transcription factor NF-kB, which is involved in the transcriptional regulation of the prosurvival genes. Specifically, these include Bcl-XL, Bcl-2, cIAP, cyclin D, and c-Myb [14].

### 3.4. Effects of Lycopene on Cell Viability

Lycopene can also carry out antitumor activity by acting at the level of cell viability [43]. According to the protocol, as regards the cell culture, all cell lines had normal growth attributes foreseen by the standard of cultures in vitro. Studies in the past have reported that 10% of water-soluble granular lycopene formulation is non-toxic. Therefore, the findings from this study referring to the 10% of WS lycopene granules are in line with other in vitro studies. The cell lines were incubated with 1, 3, and 5 µmol/L of lycopene for 24, 48, and 96 h, respectively. Lycopene was internalized in all the cells in vitro. The intracellular concentration of lycopene was highest in HT-29 and T-84 cell lines. The inhibitory effect of lycopene on cell viability was then tested at increasing concentrations. The results ascertained that the intracellular concentration of lycopene increased only after 96 h, causing a significant change in the viability of cells in six of the eight tested cell lines. These findings indicate that lycopene exerts its inhibitory effects in a cell-specific and time-dependent manner. Here, the MTT method was used to monitor cell viability. Lycopene’s effects on the cell cycle were monitored, wherein the lycopene-treated cells (1, 3, 5 µmol/L) were observed from 48 to 96 h. Thereafter, the cell percentage in various cell-cycle phases was quantified. Lycopene-treated cancer cells exhibited significant changes in their cell cycle. Specifically, there was a decrement in the G0/G1 phase as compared to that of the standard [43].

### 3.5. Effects of Lycopene on Apoptosis

Cancer is characterized by an imbalance in cellular proliferation and apoptosis. Apoptosis quantification is an important kinetic parameter of cancer cells.

Lycopene can mediate apoptosis through death receptors: Tang et al. [73] demonstrated that eicosapentaenoic acid (EPA) in conjunction with lycopene inhibits AKT (protein kinase B) and mTOR (mammalian target rapamycin) activation, improving Bax (bcl-2-like protein 4) and FasL (Fas ligand) accumulation. Consequently, cellular survival is blocked in vitro (HT-29, human colon cancer cells). Furthermore, S-allyl cysteine and lycopene cause a significant inhibition of gastric cancer development in animal models of cancer, in a combinatorial manner. This synergistic inhibitory effect is credited to their apoptotic induction activity. Together, they cause a decline in the expression of Bcl-2 while Bax and Bim are upregulated. They also reported the increased activity of caspases 8 and 3 [74]. Further studies have shown that lycopene can induce apoptosis through intrinsic pathways. For instance, apoptosis was induced in lycopene-treated mitochondrial cells (LNCaP cells) in a dose-dependent fashion. This apoptotic effect was attained due to the reduction in the cell membrane potential (mitochondria), which, in turn, prompts cytosolic Cyt c release [75]. The tumor cell lines were incubated with 3 µmol/L of lycopene for 96 h. Apoptosis was enhanced in some cell lines and after 96 h an elevation in the cell population blocked in the G2/M phase was observed. To confirm lycopene’s effect as an apoptosis-inducer, the cells were stained or DAPI-stained. The control cells possessed negligible fragmented chromatin while most of the cells treated with lycopene indicated signs of apoptosis. Precisely, these include condensed and fragmented as well as membrane vesicles. Due to the high hydrophobicity of lycopene, vehicles such as THF (tetrahydrofuran) and DMSO (dimethyl sulfoxide) were used. In this study, 10% of WS lycopene was used, which gave satisfactory and reproducible results. Furthermore, it should be considered that, in various analyses, lycopene’s effect on cell proliferation was tested within the lowest possible range, which has been already detected in human serum [43].

PDGF and IGF (growth factors) are known to improve the survival of cells as they confer protection against cellular apoptosis. Further, lycopene impedes the progress of the cell cycle from the G1 to the S phase, primarily by diminishing the cyclin D and cyclin E levels. This causes a subsequent inactivation of CDK4 and CDK2 through a reduced phosphorylation of Rb. In addition, lycopene elevates CDK inhibitor, p21, and p53 (tumor suppressor) levels, as well as minimizing SKP2 levels. Lycopene facilitates apoptosis by reducing the levels of Bcl-2 and Bcl-xl, and elevating pro-apoptotic protein levels. These include Bax, Bad, Bim, and Fas-ligand. Eventually, caspases 3, 8, and 9 are activated. Lycopene causes a blockage of the antiapoptotic signals mediated by growth factors through a direct inhibition of the link between growth factors and their receptors, or via downstream inhibition of pathways for PI3K-AKT. Lycopene also has the potential to stimulate arrest of the AKT-induced cell cycle and phosphorylation-mediated apoptosis. Finally, GSK3β, p21, p27, Bad, caspase 9, and p53 (via Mdm2) are inactivated [14].

## 4. Clinical Case Studies

Lycopene as a potential anti-cancer agent presents pharmacologically important benefits, such as abundant availability, cost-effectiveness, and minimal or no side-effects. However, substantial clinical evidence on its cancer prophylactic or therapeutic relevance is yet to be considered. Several studies and research works have been carried out that support the thesis that lycopene possesses this activity, at least in experiments carried out in cellular (cell lines) and animal models of cancer (rats).

There are few reports on lycopene and/or tomato and associated randomized clinical trials, for example; Kumar et al. conducted a phase-II randomized clinical trial in order assess the safety aspects as well as its therapeutic effects on patients with clinically localized prostate cancer. Over the period of biopsy to prostatectomy, which took approximately 30 days, 45 males were recruited and supplemented with a placebo, or 15, 30, or 45 mg of lycopene. Their results indicated that apart from the antioxidant properties of lycopene, some steroid hormone-based mechanisms may be involved [76].

Van Breemen et al. [77] reported the findings of a randomized, controlled clinical trial that included 105 males with a detected prostate-specific antigen (PSA) level above 4 ng/mL. Other diagnostics included an abnormal digital rectal or ultrasound examination. Twenty-one days prior to the prostate biopsy, patients were divided into a lycopene-treated group and a control group. The effects of lycopene on PSA level or incidence of prostate cancer were not revealed in this study. The concentration of lycopene in plasma and prostate tissues was exuberantly high in the lycopene-administered patients in comparison to the control group. However, no significant differences were found in the oxidative stress molecular markers.

Magbanua et al. also conducted a randomized clinical trial on 84 men who had a low prostate cancer risk, to assess the gene expression and biological pathways when supplemented with lycopene, fish oil, or a placebo. Their results based on exploratory analyses suggest that these micronutrients modulated candidate in vivo pathways. For instance, androgen and estrogen metabolism, Nrf2 and its mediated stress were modulated by these micronutrients [78].

Beynon et al. conducted the ProDiet randomized controlled trial on 128 men who were at risk for prostate cancer, to evaluate the roles of lycopene and green tea. They proposed that the potential of lycopene to decrease the level of pyruvate might be related to a diminished risk of prostate cancer [1].

Lane et al. conducted a Phase II randomized placebo-controlled trial on 133 men who had enhanced prostate cancer risks. They assessed the feasibility and acceptability of modifying the diet, and thus the subjects took lycopene and/or green tea as well as green tea catechins for consideration in the analysis. They found that all the tested interventions were well accepted and tolerated by all subjects while considering capsules as the preferred option. Further, they found that this male population, with a comparatively higher risk of acquiring prostate cancer, readily accepted the option of a dietary prevention strategy [2].

Lycopene’s effects in prostate cancer prevention were evaluated in three randomized controlled clinical trials involving 154 patients affected by prostatic intraepithelial neoplasia or benign prostate hyperplasia. Results indicated a decreased prostate cancer incidence despite no statistical difference being reported with the control group [79].

A meta-analysis study [80] studied tomato-based-product intake in prostate cancer risk reduction. The study was performed evaluating 21 studies involving the daily intake of one or more tomatoes, tomato derivatives, or lycopene supplements. The results indicated a limited reduction in cancer risk (11%) and were limited to a high intake of tomatoes.

A previous review reported that there is an inverse relation between tomato intake or lycopene plasma level and cancer risk at a defined anatomical site [81]. None of the reported studies indicated an increased cancer risk associated with high tomato consumption or high plasma levels of lycopene. The positive lycopene effects were reported to be stronger for prostate, lung, and stomach cancers. Moreover, data indicate a protective effect for pancreas, colorectal, esophagus, oral cavity, breast, and cervix cancers.

## 5. Conclusions

Numerous epidemiological studies have reported the beneficial health implications of carotenoid-rich fruits and vegetables in alleviating cancer-associated risks. Although there is considerable evidence for the antioxidant and, in correlation, antitumor effects of lycopene, the biological activities, underlying molecular mechanisms, and metabolism of carotenoids and lycopene remain undeciphered. To date, it is claimed that the best cure is prevention; if we can help with a healthy diet, we welcome the increased consumption of products such as tomatoes that contain substances including lycopene, obtaining all the beneficial effects mentioned so far, without negative effects. We speak of the tomato as the “emperor” of the world’s gardens, which, for its goodness and its beneficial properties has climbed the rankings of the “best” vegetables. In Italian cuisine, it has become a staple of the Mediterranean diet.
